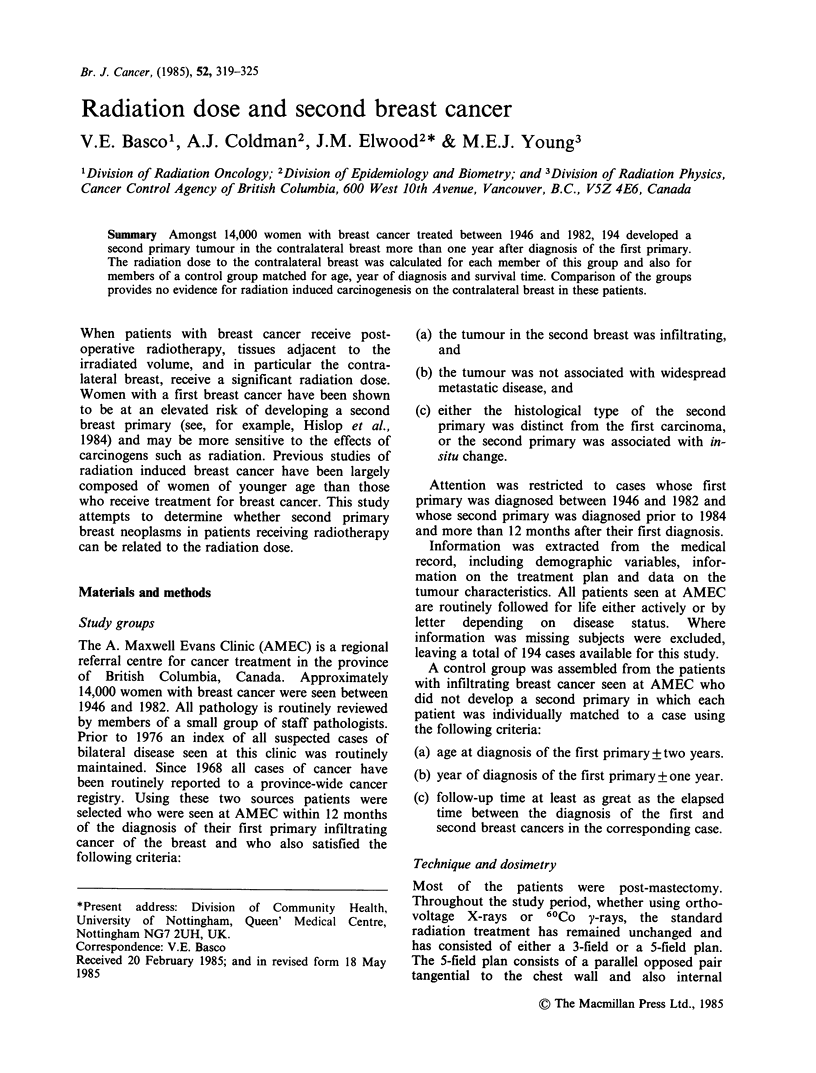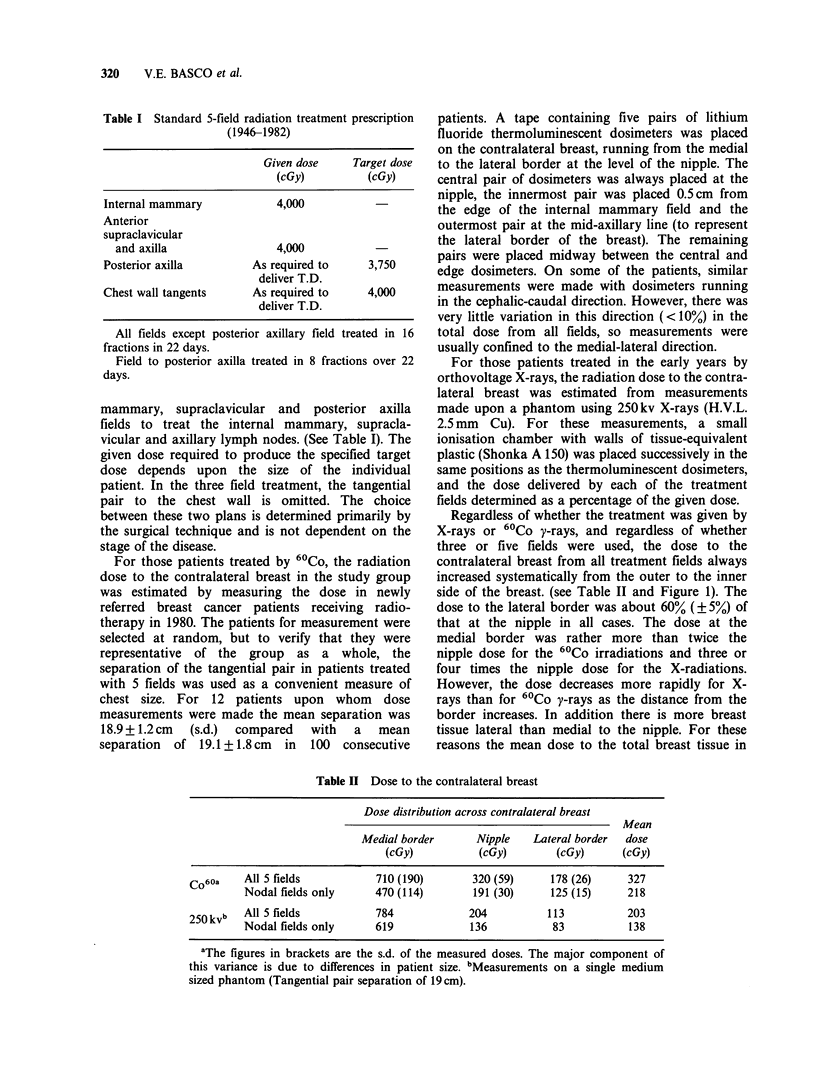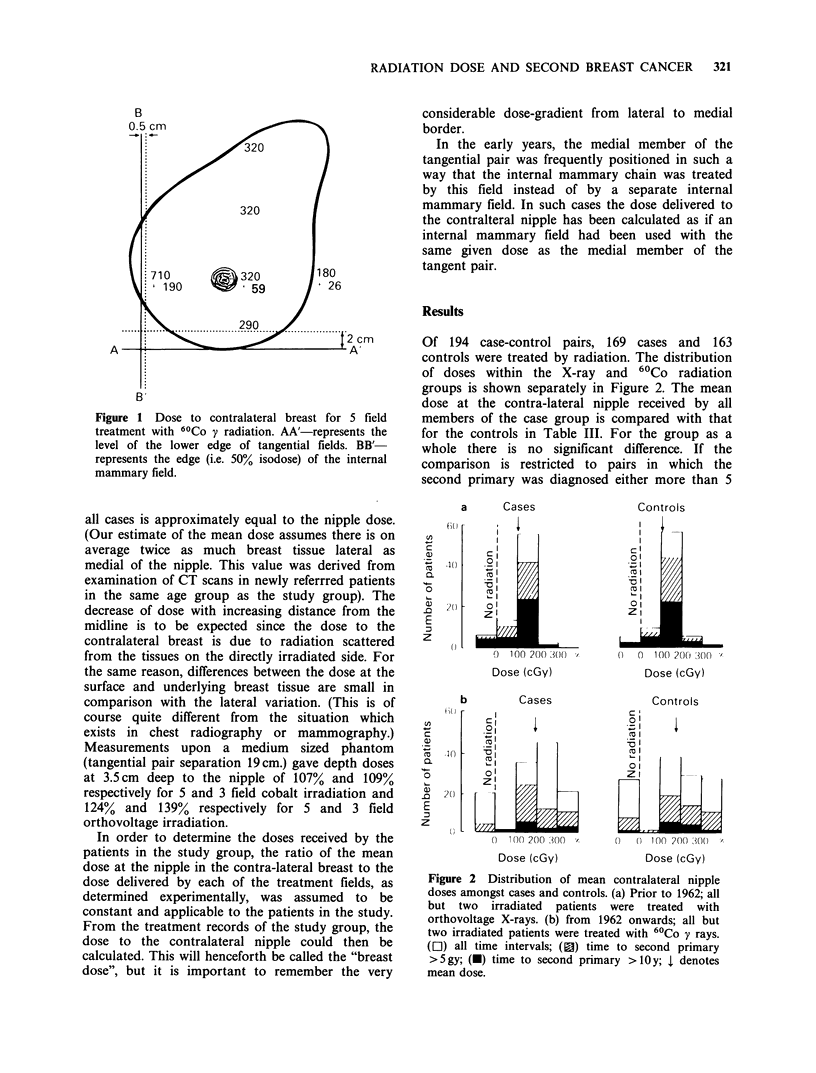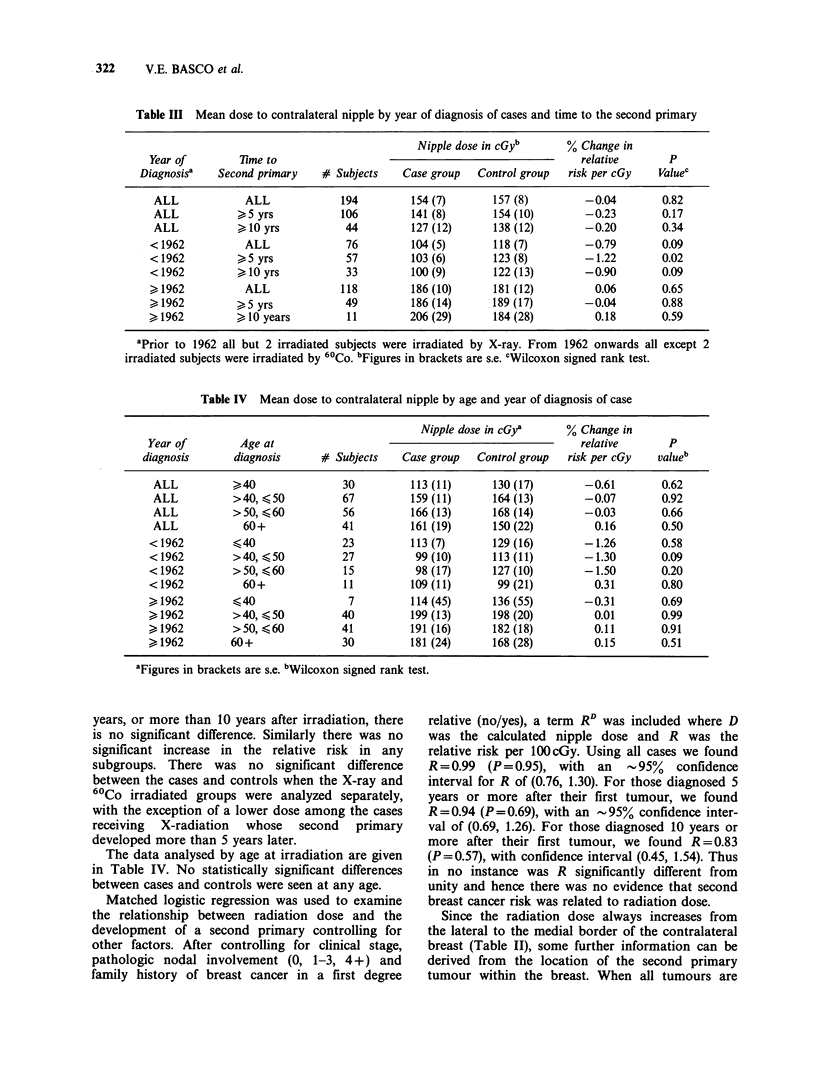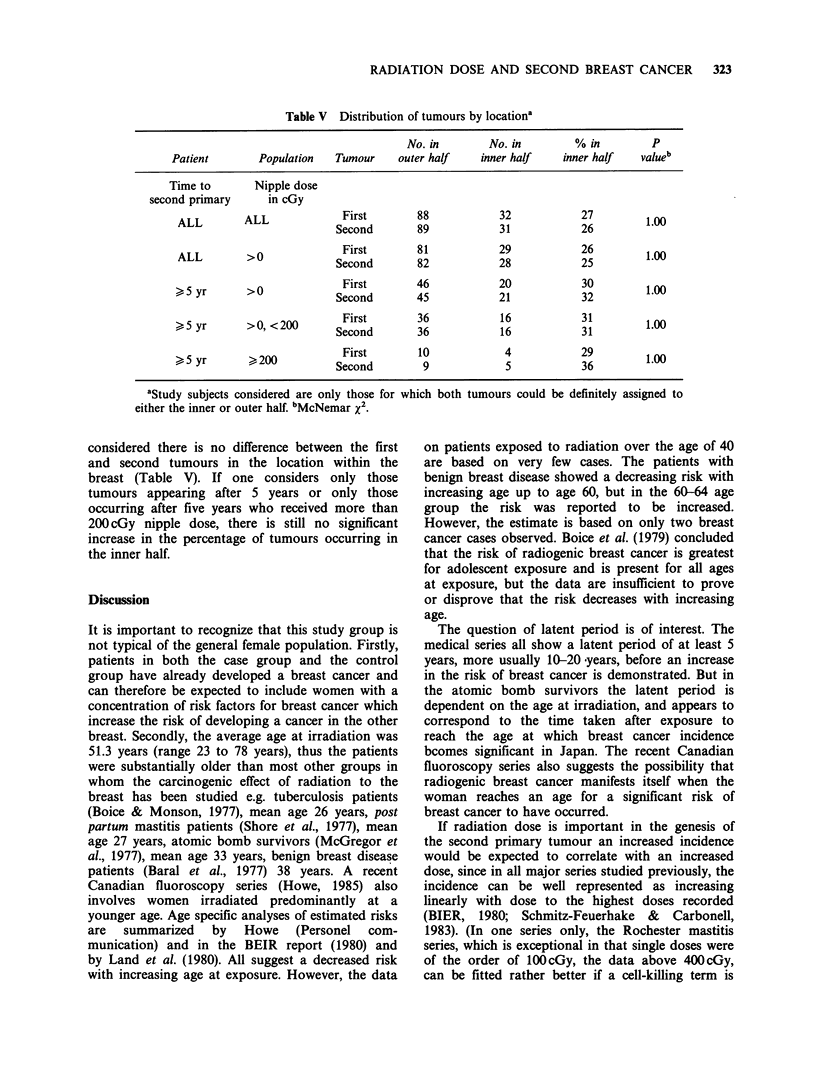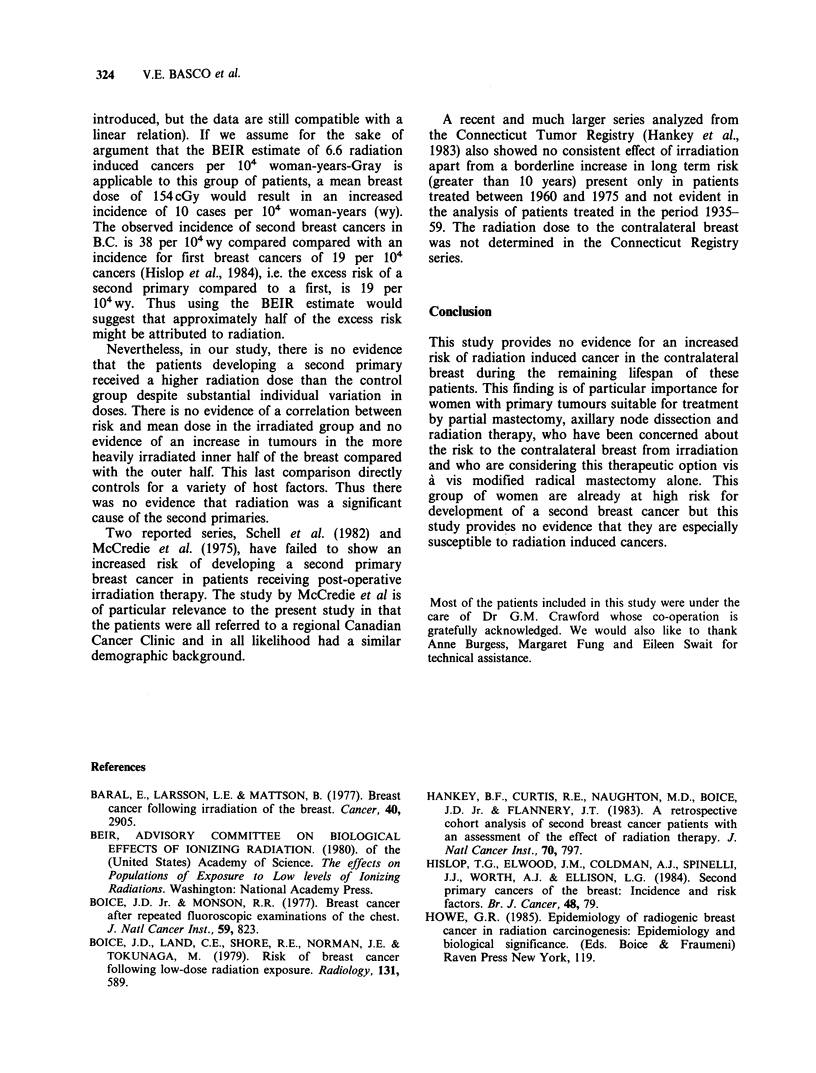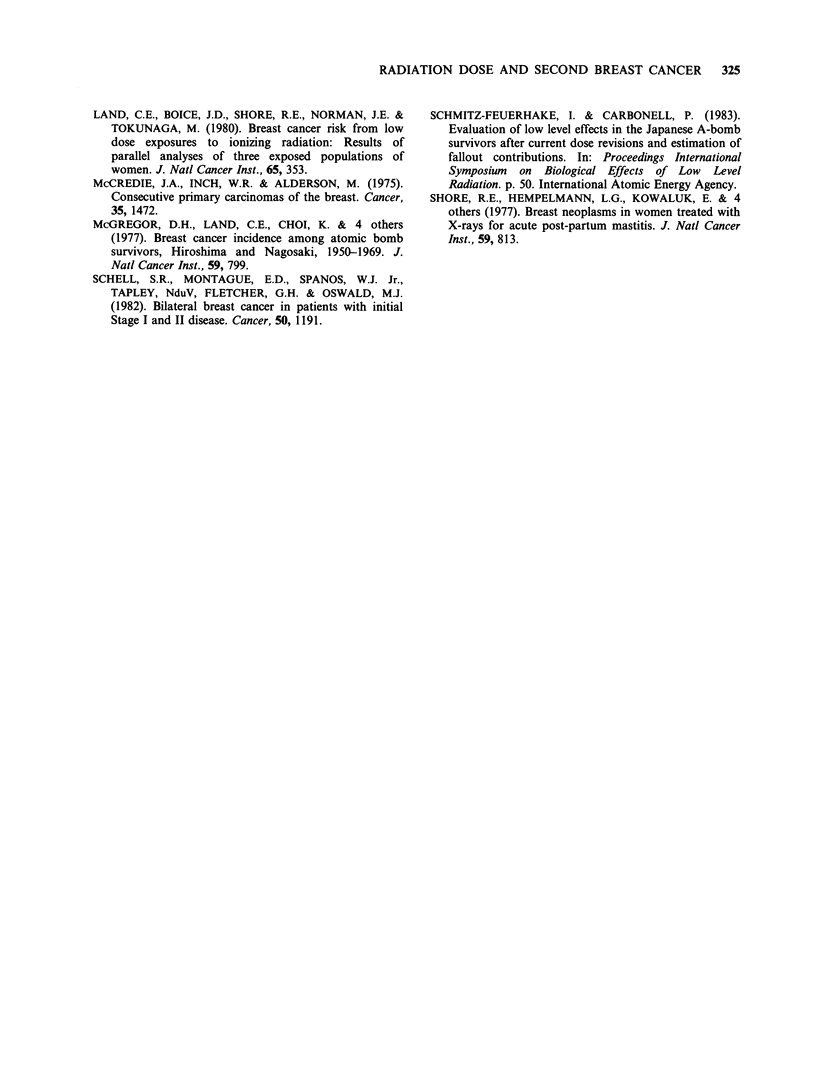# Radiation dose and second breast cancer.

**DOI:** 10.1038/bjc.1985.196

**Published:** 1985-09

**Authors:** V. E. Basco, A. J. Coldman, J. M. Elwood, M. E. Young

## Abstract

Amongst 14,000 women with breast cancer treated between 1946 and 1982, 194 developed a second primary tumour in the contralateral breast more than one year after diagnosis of the first primary. The radiation dose to the contralateral breast was calculated for each member of this group and also for members of a control group matched for age, year of diagnosis and survival time. Comparison of the groups provides no evidence for radiation induced carcinogenesis on the contralateral breast in these patients.


*Br. J. Cancer*, (1985), **52**, 319–325

### Radiation dose and second breast cancer

V.E. Basco[1], A.J. Coldman[2], J.M. Elwood[2]* & M.E.J. Young[3]

[1]*Division of Radiation Oncology;* [2]*Division of Epidemiology and Biometry; and* [3]*Division of Radiation Physics, Cancer Control Agency of British Columbia, 600 West 10th Avenue, Vancouver, B.C., V5Z 4E6, Canada*

**Summary** Amongst 14,000 women with breast cancer treated between 1946 and 1982, 194 developed a second primary tumour in the contralateral breast more than one year after diagnosis of the first primary. The radiation dose to the contralateral breast was calculated for each member of this group and also for members of a control group matched for age, year of diagnosis and survival time. Comparison of the groups provides no evidence for radiation induced carcinogenesis on the contralateral breast in these patients.

When patients with breast cancer receive post-operative radiotherapy, tissues adjacent to the irradiated volume, and in particular the contralateral breast, receive a significant radiation dose. Women with a first breast cancer have been shown to be at an elevated risk of developing a second breast primary (see, for example, Hislop *et al.*, 1984) and may be more sensitive to the effects of carcinogens such as radiation. Previous studies of radiation induced breast cancer have been largely composed of women of younger age than those who receive treatment for breast cancer. This study attempts to determine whether second primary breast neoplasms in patients receiving radiotherapy can be related to the radiation dose.

### Materials and methods

### *Study groups*

The A. Maxwell Evans Clinic (AMEC) is a regional referral centre for cancer treatment in the province of British Columbia, Canada. Approximately 14,000 women with breast cancer were seen between 1946 and 1982. All pathology is routinely reviewed by members of a small group of staff pathologists. Prior to 1976 an index of all suspected cases of bilateral disease seen at this clinic was routinely maintained. Since 1968 all cases of cancer have been routinely reported to a province-wide cancer registry. Using these two sources patients were selected who were seen at AMEC within 12 months of the diagnosis of their first primary infiltrating cancer of the breast and who also satisfied the following criteria:

(a) the tumour in the second breast was infiltrating, and

(b) the tumour was not associated with widespread metastatic disease, and

(c) either the histological type of the second primary was distinct from the first carcinoma, or the second primary was associated with *in-situ* change.

Attention was restricted to cases whose first primary was diagnosed between 1946 and 1982 and whose second primary was diagnosed prior to 1984 and more than 12 months after their first diagnosis.

Information was extracted from the medical record, including demographic variables, information on the treatment plan and data on the tumour characteristics. All patients seen at AMEC are routinely followed for life either actively or by letter depending on disease status. Where information was missing subjects were excluded, leaving a total of 194 cases available for this study.

A control group was assembled from the patients with infiltrating breast cancer seen at AMEC who did not develop a second primary in which each patient was individually matched to a case using the following criteria:

(a) age at diagnosis of the first primary $\pm$ two years.

(b) year of diagnosis of the first primary $\pm$ one year.

(c) follow-up time at least as great as the elapsed time between the diagnosis of the first and second breast cancers in the corresponding case.

### *Technique and dosimetry*

Most of the patients were post-mastectomy. Throughout the study period, whether using orthovoltage X-rays or $^{60}Co$ $\gamma$-rays, the standard radiation treatment has remained unchanged and has consisted of either a 3-field or a 5-field plan. The 5-field plan consists of a parallel opposed pair tangential to the chest wall and also internal

*Present address: Division of Community Health, University of Nottingham, Queen' Medical Centre, Nottingham NG7 2UH, UK.

Correspondence: V.E. Basco

Received 20 February 1985; and in revised form 18 May 1985

© The Macmillan Press Ltd., 1985

**Table I** Standard 5-field radiation treatment prescription (1946–1982)

| | *Given dose (cGy)* | *Target dose (cGy)* |
|---|---|---|
| Internal mammary | 4,000 | — |
| Anterior supraclavicular and axilla | 4,000 | — |
| Posterior axilla | As required to deliver T.D. | 3,750 |
| Chest wall tangents | As required to deliver T.D. | 4,000 |

All fields except posterior axillary field treated in 16 fractions in 22 days.

Field to posterior axilla treated in 8 fractions over 22 days.

mammary, supraclavicular and posterior axilla fields to treat the internal mammary, supraclavicular and axillary lymph nodes. (See Table I). The given dose required to produce the specified target dose depends upon the size of the individual patient. In the three field treatment, the tangential pair to the chest wall is omitted. The choice between these two plans is determined primarily by the surgical technique and is not dependent on the stage of the disease.

For those patients treated by $^{60}$Co, the radiation dose to the contralateral breast in the study group was estimated by measuring the dose in newly referred breast cancer patients receiving radiotherapy in 1980. The patients for measurement were selected at random, but to verify that they were representative of the group as a whole, the separation of the tangential pair in patients treated with 5 fields was used as a convenient measure of chest size. For 12 patients upon whom dose measurements were made the mean separation was $18.9 \pm 1.2$ cm (s.d.) compared with a mean separation of $19.1 \pm 1.8$ cm in 100 consecutive patients. A tape containing five pairs of lithium fluoride thermoluminescent dosimeters was placed on the contralateral breast, running from the medial to the lateral border at the level of the nipple. The central pair of dosimeters was always placed at the nipple, the innermost pair was placed 0.5 cm from the edge of the internal mammary field and the outermost pair at the mid-axillary line (to represent the lateral border of the breast). The remaining pairs were placed midway between the central and edge dosimeters. On some of the patients, similar measurements were made with dosimeters running in the cephalic-caudal direction. However, there was very little variation in this direction ($<10\%$) in the total dose from all fields, so measurements were usually confined to the medial-lateral direction.

For those patients treated in the early years by orthovoltage X-rays, the radiation dose to the contralateral breast was estimated from measurements made upon a phantom using 250 kv X-rays (H.V.L. 2.5 mm Cu). For these measurements, a small ionisation chamber with walls of tissue-equivalent plastic (Shonka A 150) was placed successively in the same positions as the thermoluminescent dosimeters, and the dose delivered by each of the treatment fields determined as a percentage of the given dose.

Regardless of whether the treatment was given by X-rays or $^{60}$Co $\gamma$-rays, and regardless of whether three or five fields were used, the dose to the contralateral breast from all treatment fields always increased systematically from the outer to the inner side of the breast. (see Table II and Figure 1). The dose to the lateral border was about 60% ($\pm 5\%$) of that at the nipple in all cases. The dose at the medial border was rather more than twice the nipple dose for the $^{60}$Co irradiations and three or four times the nipple dose for the X-radiations. However, the dose decreases more rapidly for X-rays than for $^{60}$Co $\gamma$-rays as the distance from the border increases. In addition there is more breast tissue lateral than medial to the nipple. For these reasons the mean dose to the total breast tissue in

**Table II** Dose to the contralateral breast

| | | *Dose distribution across contralateral breast* | | | *Mean dose (cGy)* |
|---|---|---|---|---|---|
| | | *Medial border (cGy)* | *Nipple (cGy)* | *Lateral border (cGy)* | |
| $Co^{60a}$ | All 5 fields | 710 (190) | 320 (59) | 178 (26) | 327 |
| | Nodal fields only | 470 (114) | 191 (30) | 125 (15) | 218 |
| 250 kv[b] | All 5 fields | 784 | 204 | 113 | 203 |
| | Nodal fields only | 619 | 136 | 83 | 138 |

[a]The figures in brackets are the s.d. of the measured doses. The major component of this variance is due to differences in patient size. [b]Measurements on a single medium sized phantom (Tangential pair separation of 19 cm).

**Figure 1** Dose to contralateral breast for 5 field treatment with $^{60}Co$ $\gamma$ radiation. AA′—represents the level of the lower edge of tangential fields. BB′—represents the edge (i.e. 50% isodose) of the internal mammary field.

all cases is approximately equal to the nipple dose. (Our estimate of the mean dose assumes there is on average twice as much breast tissue lateral as medial of the nipple. This value was derived from examination of CT scans in newly referrred patients in the same age group as the study group). The decrease of dose with increasing distance from the midline is to be expected since the dose to the contralateral breast is due to radiation scattered from the tissues on the directly irradiated side. For the same reason, differences between the dose at the surface and underlying breast tissue are small in comparison with the lateral variation. (This is of course quite different from the situation which exists in chest radiography or mammography.) Measurements upon a medium sized phantom (tangential pair separation 19 cm.) gave depth doses at 3.5 cm deep to the nipple of 107% and 109% respectively for 5 and 3 field cobalt irradiation and 124% and 139% respectively for 5 and 3 field orthovoltage irradiation.

In order to determine the doses received by the patients in the study group, the ratio of the mean dose at the nipple in the contra-lateral breast to the dose delivered by each of the treatment fields, as determined experimentally, was assumed to be constant and applicable to the patients in the study. From the treatment records of the study group, the dose to the contralateral nipple could then be calculated. This will henceforth be called the "breast dose", but it is important to remember the very considerable dose-gradient from lateral to medial border.

In the early years, the medial member of the tangential pair was frequently positioned in such a way that the internal mammary chain was treated by this field instead of by a separate internal mammary field. In such cases the dose delivered to the contralteral nipple has been calculated as if an internal mammary field had been used with the same given dose as the medial member of the tangent pair.

### Results

Of 194 case-control pairs, 169 cases and 163 controls were treated by radiation. The distribution of doses within the X-ray and $^{60}Co$ radiation groups is shown separately in Figure 2. The mean dose at the contra-lateral nipple received by all members of the case group is compared with that for the controls in Table III. For the group as a whole there is no significant difference. If the comparison is restricted to pairs in which the second primary was diagnosed either more than 5

**Figure 2** Distribution of mean contralateral nipple doses amongst cases and controls. (a) Prior to 1962; all but two irradiated patients were treated with orthovoltage X-rays. (b) from 1962 onwards; all but two irradiated patients were treated with $^{60}Co$ $\gamma$ rays. (□) all time intervals; (▨) time to second primary >5 gy; (■) time to second primary >10 y; ↓ denotes mean dose.

**Table III** Mean dose to contralateral nipple by year of diagnosis of cases and time to the second primary

| Year of Diagnosis[a] | Time to Second primary | # Subjects | Nipple dose in cGy[b] Case group | Nipple dose in cGy[b] Control group | % Change in relative risk per cGy | P Value[c] |
|---|---|---|---|---|---|---|
| ALL | ALL | 194 | 154 (7) | 157 (8) | −0.04 | 0.82 |
| ALL | ≥5 yrs | 106 | 141 (8) | 154 (10) | −0.23 | 0.17 |
| ALL | ≥10 yrs | 44 | 127 (12) | 138 (12) | −0.20 | 0.34 |
| <1962 | ALL | 76 | 104 (5) | 118 (7) | −0.79 | 0.09 |
| <1962 | ≥5 yrs | 57 | 103 (6) | 123 (8) | −1.22 | 0.02 |
| <1962 | ≥10 yrs | 33 | 100 (9) | 122 (13) | −0.90 | 0.09 |
| ≥1962 | ALL | 118 | 186 (10) | 181 (12) | 0.06 | 0.65 |
| ≥1962 | ≥5 yrs | 49 | 186 (14) | 189 (17) | −0.04 | 0.88 |
| ≥1962 | ≥10 years | 11 | 206 (29) | 184 (28) | 0.18 | 0.59 |

[a]Prior to 1962 all but 2 irradiated subjects were irradiated by X-ray. From 1962 onwards all except 2 irradiated subjects were irradiated by $^{60}$Co. [b]Figures in brackets are s.e. [c]Wilcoxon signed rank test.

**Table IV** Mean dose to contralateral nipple by age and year of diagnosis of case

| Year of diagnosis | Age at diagnosis | # Subjects | Nipple dose in cGy[a] Case group | Nipple dose in cGy[a] Control group | % Change in relative risk per cGy | P value[b] |
|---|---|---|---|---|---|---|
| ALL | ≥40 | 30 | 113 (11) | 130 (17) | −0.61 | 0.62 |
| ALL | >40, ≤50 | 67 | 159 (11) | 164 (13) | −0.07 | 0.92 |
| ALL | >50, ≤60 | 56 | 166 (13) | 168 (14) | −0.03 | 0.66 |
| ALL | 60+ | 41 | 161 (19) | 150 (22) | 0.16 | 0.50 |
| <1962 | ≤40 | 23 | 113 (7) | 129 (16) | −1.26 | 0.58 |
| <1962 | >40, ≤50 | 27 | 99 (10) | 113 (11) | −1.30 | 0.09 |
| <1962 | >50, ≤60 | 15 | 98 (17) | 127 (10) | −1.50 | 0.20 |
| <1962 | 60+ | 11 | 109 (11) | 99 (21) | 0.31 | 0.80 |
| ≥1962 | ≤40 | 7 | 114 (45) | 136 (55) | −0.31 | 0.69 |
| ≥1962 | >40, ≤50 | 40 | 199 (13) | 198 (20) | 0.01 | 0.99 |
| ≥1962 | >50, ≤60 | 41 | 191 (16) | 182 (18) | 0.11 | 0.91 |
| ≥1962 | 60+ | 30 | 181 (24) | 168 (28) | 0.15 | 0.51 |

[a]Figures in brackets are s.e. [b]Wilcoxon signed rank test.

years, or more than 10 years after irradiation, there is no significant difference. Similarly there was no significant increase in the relative risk in any subgroups. There was no significant difference between the cases and controls when the X-ray and $^{60}$Co irradiated groups were analyzed separately, with the exception of a lower dose among the cases receiving X-radiation whose second primary developed more than 5 years later.

The data analysed by age at irradiation are given in Table IV. No statistically significant differences between cases and controls were seen at any age.

Matched logistic regression was used to examine the relationship between radiation dose and the development of a second primary controlling for other factors. After controlling for clinical stage, pathologic nodal involvement (0, 1–3, 4+) and family history of breast cancer in a first degree relative (no/yes), a term $R^D$ was included where $D$ was the calculated nipple dose and $R$ was the relative risk per 100 cGy. Using all cases we found $R=0.99$ ($P=0.95$), with an ~95% confidence interval for $R$ of (0.76, 1.30). For those diagnosed 5 years or more after their first tumour, we found $R=0.94$ ($P=0.69$), with an ~95% confidence interval of (0.69, 1.26). For those diagnosed 10 years or more after their first tumour, we found $R=0.83$ ($P=0.57$), with confidence interval (0.45, 1.54). Thus in no instance was $R$ significantly different from unity and hence there was no evidence that second breast cancer risk was related to radiation dose.

Since the radiation dose always increases from the lateral to the medial border of the contralateral breast (Table II), some further information can be derived from the location of the second primary tumour within the breast. When all tumours are

**Table V** Distribution of tumours by location[a]

| *Patient* | *Population* | *Tumour* | *No. in outer half* | *No. in inner half* | *% in inner half* | *P value*[b] |
|---|---|---|---|---|---|---|
| Time to second primary | Nipple dose in cGy | | | | | |
| ALL | ALL | First | 88 | 32 | 27 | 1.00 |
| | | Second | 89 | 31 | 26 | |
| ALL | >0 | First | 81 | 29 | 26 | 1.00 |
| | | Second | 82 | 28 | 25 | |
| ≥5 yr | >0 | First | 46 | 20 | 30 | 1.00 |
| | | Second | 45 | 21 | 32 | |
| ≥5 yr | >0, <200 | First | 36 | 16 | 31 | 1.00 |
| | | Second | 36 | 16 | 31 | |
| ≥5 yr | ≥200 | First | 10 | 4 | 29 | 1.00 |
| | | Second | 9 | 5 | 36 | |

[a]Study subjects considered are only those for which both tumours could be definitely assigned to either the inner or outer half. [b]McNemar $\chi^2$.

considered there is no difference between the first and second tumours in the location within the breast (Table V). If one considers only those tumours appearing after 5 years or only those occurring after five years who received more than 200 cGy nipple dose, there is still no significant increase in the percentage of tumours occurring in the inner half.

### Discussion

It is important to recognize that this study group is not typical of the general female population. Firstly, patients in both the case group and the control group have already developed a breast cancer and can therefore be expected to include women with a concentration of risk factors for breast cancer which increase the risk of developing a cancer in the other breast. Secondly, the average age at irradiation was 51.3 years (range 23 to 78 years), thus the patients were substantially older than most other groups in whom the carcinogenic effect of radiation to the breast has been studied e.g. tuberculosis patients (Boice & Monson, 1977), mean age 26 years, *post partum* mastitis patients (Shore *et al.*, 1977), mean age 27 years, atomic bomb survivors (McGregor *et al.*, 1977), mean age 33 years, benign breast disease patients (Baral *et al.*, 1977) 38 years. A recent Canadian fluoroscopy series (Howe, 1985) also involves women irradiated predominantly at a younger age. Age specific analyses of estimated risks are summarized by Howe (Personel communication) and in the BEIR report (1980) and by Land *et al.* (1980). All suggest a decreased risk with increasing age at exposure. However, the data on patients exposed to radiation over the age of 40 are based on very few cases. The patients with benign breast disease showed a decreasing risk with increasing age up to age 60, but in the 60–64 age group the risk was reported to be increased. However, the estimate is based on only two breast cancer cases observed. Boice *et al.* (1979) concluded that the risk of radiogenic breast cancer is greatest for adolescent exposure and is present for all ages at exposure, but the data are insufficient to prove or disprove that the risk decreases with increasing age.

The question of latent period is of interest. The medical series all show a latent period of at least 5 years, more usually 10–20 years, before an increase in the risk of breast cancer is demonstrated. But in the atomic bomb survivors the latent period is dependent on the age at irradiation, and appears to correspond to the time taken after exposure to reach the age at which breast cancer incidence bcomes significant in Japan. The recent Canadian fluoroscopy series also suggests the possibility that radiogenic breast cancer manifests itself when the woman reaches an age for a significant risk of breast cancer to have occurred.

If radiation dose is important in the genesis of the second primary tumour an increased incidence would be expected to correlate with an increased dose, since in all major series studied previously, the incidence can be well represented as increasing linearly with dose to the highest doses recorded (BIER, 1980; Schmitz-Feuerhake & Carbonell, 1983). (In one series only, the Rochester mastitis series, which is exceptional in that single doses were of the order of 100 cGy, the data above 400 cGy, can be fitted rather better if a cell-killing term is introduced, but the data are still compatible with a linear relation). If we assume for the sake of argument that the BEIR estimate of 6.6 radiation induced cancers per $10^4$ woman-years-Gray is applicable to this group of patients, a mean breast dose of 154 cGy would result in an increased incidence of 10 cases per $10^4$ woman-years (wy). The observed incidence of second breast cancers in B.C. is 38 per $10^4$ wy compared compared with an incidence for first breast cancers of 19 per $10^4$ cancers (Hislop *et al.*, 1984), i.e. the excess risk of a second primary compared to a first, is 19 per $10^4$ wy. Thus using the BEIR estimate would suggest that approximately half of the excess risk might be attributed to radiation.

Nevertheless, in our study, there is no evidence that the patients developing a second primary received a higher radiation dose than the control group despite substantial individual variation in doses. There is no evidence of a correlation between risk and mean dose in the irradiated group and no evidence of an increase in tumours in the more heavily irradiated inner half of the breast compared with the outer half. This last comparison directly controls for a variety of host factors. Thus there was no evidence that radiation was a significant cause of the second primaries.

Two reported series, Schell *et al.* (1982) and McCredie *et al.* (1975), have failed to show an increased risk of developing a second primary breast cancer in patients receiving post-operative irradiation therapy. The study by McCredie *et al* is of particular relevance to the present study in that the patients were all referred to a regional Canadian Cancer Clinic and in all likelihood had a similar demographic background.

A recent and much larger series analyzed from the Connecticut Tumor Registry (Hankey *et al.*, 1983) also showed no consistent effect of irradiation apart from a borderline increase in long term risk (greater than 10 years) present only in patients treated between 1960 and 1975 and not evident in the analysis of patients treated in the period 1935–59. The radiation dose to the contralateral breast was not determined in the Connecticut Registry series.

### Conclusion

This study provides no evidence for an increased risk of radiation induced cancer in the contralateral breast during the remaining lifespan of these patients. This finding is of particular importance for women with primary tumours suitable for treatment by partial mastectomy, axillary node dissection and radiation therapy, who have been concerned about the risk to the contralateral breast from irradiation and who are considering this therapeutic option vis à vis modified radical mastectomy alone. This group of women are already at high risk for development of a second breast cancer but this study provides no evidence that they are especially susceptible to radiation induced cancers.

Most of the patients included in this study were under the care of Dr G.M. Crawford whose co-operation is gratefully acknowledged. We would also like to thank Anne Burgess, Margaret Fung and Eileen Swait for technical assistance.

### References

BARAL, E., LARSSON, L.E. & MATTSON, B. (1977). Breast cancer following irradiation of the breast. *Cancer*, **40**, 2905.

BEIR, ADVISORY COMMITTEE ON BIOLOGICAL EFFECTS OF IONIZING RADIATION. (1980). of the (United States) Academy of Science. *The effects on Populations of Exposure to Low levels of Ionizing Radiations*. Washington: National Academy Press.

BOICE, J.D. Jr. & MONSON, R.R. (1977). Breast cancer after repeated fluoroscopic examinations of the chest. *J. Natl Cancer Inst.*, **59**, 823.

BOICE, J.D., LAND, C.E., SHORE, R.E., NORMAN, J.E. & TOKUNAGA, M. (1979). Risk of breast cancer following low-dose radiation exposure. *Radiology*, **131**, 589.

HANKEY, B.F., CURTIS, R.E., NAUGHTON, M.D., BOICE, J.D. Jr. & FLANNERY, J.T. (1983). A retrospective cohort analysis of second breast cancer patients with an assessment of the effect of radiation therapy. *J. Natl Cancer Inst.*, **70**, 797.

HISLOP, T.G., ELWOOD, J.M., COLDMAN, A.J., SPINELLI, J.J., WORTH, A.J. & ELLISON, L.G. (1984). Second primary cancers of the breast: Incidence and risk factors. *Br. J. Cancer*, **48**, 79.

HOWE, G.R. (1985). Epidemiology of radiogenic breast cancer in radiation carcinogenesis: Epidemiology and biological significance. (Eds. Boice & Fraumeni) Raven Press New York, 119.

LAND, C.E., BOICE, J.D., SHORE, R.E., NORMAN, J.E. & TOKUNAGA, M. (1980). Breast cancer risk from low dose exposures to ionizing radiation: Results of parallel analyses of three exposed populations of women. *J. Natl Cancer Inst.*, **65**, 353.

McCREDIE, J.A., INCH, W.R. & ALDERSON, M. (1975). Consecutive primary carcinomas of the breast. *Cancer*, **35**, 1472.

McGREGOR, D.H., LAND, C.E., CHOI, K. & 4 others (1977). Breast cancer incidence among atomic bomb survivors, Hiroshima and Nagosaki, 1950–1969. *J. Natl Cancer Inst.*, **59**, 799.

SCHELL, S.R., MONTAGUE, E.D., SPANOS, W.J. Jr., TAPLEY, NduV, FLETCHER, G.H. & OSWALD, M.J. (1982). Bilateral breast cancer in patients with initial Stage I and II disease. *Cancer*, **50**, 1191.

SCHMITZ-FEUERHAKE, I. & CARBONELL, P. (1983). Evaluation of low level effects in the Japanese A-bomb survivors after current dose revisions and estimation of fallout contributions. In: *Proceedings International Symposium on Biological Effects of Low Level Radiation*. p. 50. International Atomic Energy Agency.

SHORE, R.E., HEMPELMANN, L.G., KOWALUK, E. & 4 others (1977). Breast neoplasms in women treated with X-rays for acute post-partum mastitis. *J. Natl Cancer Inst.*, **59**, 813.